# Self-Rated Health among Urban Adolescents: The Roles of Age, Gender, and Their Associated Factors

**DOI:** 10.1371/journal.pone.0132254

**Published:** 2015-07-15

**Authors:** Adriana Lúcia Meireles, César Coelho Xavier, Amanda Cristina de Souza Andrade, Fernando Augusto Proietti, Waleska Teixeira Caiaffa

**Affiliations:** 1 Nutrition, Faculdade de Ciências da Saúde do Trairi, Federal University of Rio Grande do Norte, Natal, Rio Grande do Norte, Brazil; 2 Observatory for Urban Health of Belo Horizonte, School of Medicine, Federal University of Minas Gerais, Belo Horizonte, Minas Gerais, Brazil; 3 Medicine, Faculdade de Saúde e Ecologia Humana, Vespasiano, Minas Gerais, Brazil; David Geffen School of Medicine at UCLA, UNITED STATES

## Abstract

Health status is often analyzed in population surveys. Self-rated health (SRH) is a single-item summary measure of the perception of one’s health. In Brazil, studies on the SRH of adolescents remain scarce, especially those aiming to understand the domains that compose this construct. Therefore, the aim of this study is to determine the prevalence of poor SRH and its associated factors among 11- to 13-year-olds and 14- to 17-year-olds living in a large urban center in Brazil. This cross-sectional study was conducted using a household survey across Belo Horizonte that included 1,042 adolescents. Stratified logistic regression models were used for each age group to assess the associations between worse SRH and the following variables: socio-demographic, social and family support, lifestyles, psychological health, and anthropometry. Approximately 11% (95% CIs = 8.7–13.6) of the studied adolescents rated their health as poor, and SHR decreased with age among males and females. This trend was more pronounced among girls (from 6.9% among 11- to 13-year-old girls to 16.9% among 14- to 17-year-old girls) than boys (from 8.3% among 11- to 13-year-old boys to 11% among 14- to 17-year-old boys). Worse SRH was associated with family support (as assessed by the absence of parent-adolescent conversations; odds ratio [OR] = 3.5 among 11- to 13-year-olds), family structure (OR = 2.8 among 14- to 17-year-olds), and argument reporting (OR = 8.2 among 14- to 17-year-olds). Among older adolescents, the consumption of fruit fewer than five times per week (OR = 2.4), life dissatisfaction (OR = 2.8), underweight status (OR = 6.7), and overweight status (OR = 2.7) were associated with poor SRH. As adolescents age, their universe expands from their relationship with their parents to include more complex issues, such as their lifestyles and life satisfaction. Therefore, these results suggest the importance of evaluating SRH across adolescent age groups and demonstrate the influence of the family environment (in addition to other factors) on negative health assessments, particularly among 14- to 17-year-olds.

## Introduction

Health status is often analyzed in population surveys. Self-rated health (SRH), a single-item summary measure of the perception of one’s health, is universally considered appropriate to assess the health status of adults [1] and the elderly [2]. Although between-study variability exists with regard to the framing of the question and its response options, respondents most often rate their health using a five-point scale, ranging from “very good” to “very poor”.

Studies on the use of this summary measure and its determining factors have been widely performed among adults, and the prevalence of poor SRH ranges from 25% to 40% in urban populations [3,4,5]. Among adolescents, the use of this summary measure is increasing [6,7,8,9], and the prevalence of poor SRH ranges from 10% to 25% [7,8,10,11]. From a public health perspective, the prevalence of poor SRH in adolescents seems high because this age group usually has low morbidity rates [8]. Haugland et al. (2001) suggested that health perception deteriorates during adolescence, when numerous youths report subjective health concerns and complaints [12].

Studies regarding the possible constructs involved in the understanding of SRH have been conducted [1,13], suggesting that, among adolescents, this measure is a multidimensional construct that reflects domains beyond physical health, involving family, social and peer support, psychological well-being, socio-demographic, behavior and lifestyle factors [5,7,14]. In Brazil, however, studies on the SRH of adolescents remain scarce, especially those aiming to understand the domains that compose this construct. Recently, two Brazilian studies assessed SRH among adolescents and the prevalence and factors associated with negative perceptions of health. However, both studies only interviewed 14- to 19-year-olds in schools. The first study was conducted among students in Santa Catarina [9], and the second study, in João Pessoa [15].

The individual characteristics and experiences, combined with the influence of environmental factors, modulate the development during adolescence, including health development. The puberty period is the phase of human development during which physical growth and sexual maturation occur in an accelerated fashion, and the underlying biological processes result in physical changes that have clear intellectual, emotional, social and behavioral implications, many of which result in health-related outcomes [16]. The onset of puberty varies among individuals; thus, the rate of physical changes varies correspondingly. These changes in body appearance can pose a major adaptation challenge for teens. Additionally, as teens become more aware of their bodies during puberty, affecting how they feel about themselves and relate to others, this period of change influences their social behavior and health perception [16].

Given that adolescence is a developmental stage characterized by constant physical and psychosocial changes and the formation of the cognitive concept of health [6,8], health status perceptions and their determinants might be expected to change throughout this age period [1]. Although some studies have shown that older adolescents are more likely to have poor SRH compared with younger adolescents [10,12,17], our understanding of the possible factors associated with this construct remains incomplete, especially in countries in the southern hemisphere.

Furthermore, because the perceived health status formed during adolescence (regardless of specific age group) might persist into adulthood, it is important to identify and to understand the determinants of SRH among youths (i.e., How do adolescents assess their health across age groups? What are the factors associated with poor SRH among adolescents across age groups?). Therefore, this study seeks to test the hypothesis that there were differences between younger and older adolescents in relationships between potential predictors for poor SRH.

## Methods

The present study is part of the population-based household survey, the “Health in Beagá Study”, conducted by the Belo Horizonte Observatory for Urban Health (OSUBH) at the Universidade Federal de Minas Gerais (UFMG) between 2008 and 2009 across two of the nine health districts of Belo Horizonte, Minas Gerais (MG). These districts account for 22.4% of the city’s population of 2,375,151 inhabitants [18], which is characterized by a broad socioeconomic heterogeneity [19, 20].

The sample was selected using stratified three-stage cluster sampling, including census tracts as the first level, households as the second level and residents as the third level. The sample strata were defined according to the Health Vulnerability Index [12], an index created by combining social, demographic, economic and health indicators from each census tract. The index was developed by the Municipal Health Department of Belo Horizonte [19] and was geocoded by census tracts. Census tracts are defined by the Brazilian Institute of Geography and Statistics and include an average of 1,000 residents each.

In the first stage, 149 census tracts were selected from a total of 588 census tracts in the sampling frame. In the second stage, 7,942 households were initially eligible, using a sampling frame from the municipality. After deleting vacant lots, institutional and commercial buildings and eligible participants who were not found after three visits to their homes, 5,171 households remained eligible. The refusal rate was approximately 12.1%, resulting in a study sample of 4,048 households. In the third stage, one adolescent aged 11–17 years of age and an adult 18 years or older were randomly selected to participate within each sampled household [13].

Of the 4,048 households sampled, 1,197 had eligible adolescents who were invited to participate. Of these adolescents, 1,042 participated in the study. Losses (12,9%) did not significantly differ by gender or age, and they occurred because of refusal to participate.

### Instruments

The information was gathered using self-administered and confidential instruments composed of questions regarding educational, well-being, social and family contexts, physical activity, eating habit markers, and subjective well-being evaluations, including psychological well-being, life satisfaction, and body satisfaction. The collection instruments were prepared by OSUBH based on Brazilian [21] and international [23] studies. All instruments were pre-coded and pre-tested in a pilot study. An anthropometric assessment was performed to measure participants’ heights and weights using a TANITA BC-553 scale and an anthropometer according to recommendations of the World Health Organization [24].

### Dependent variable

The dependent variable was SRH defined by the question, “In general, do you consider your health: very good, good, fair, poor, or very poor?” Subsequently, answers of fair, poor, and very poor health were combined into the category “poor SRH”, and reports of good and very good health were combined into the category “good SRH” for comparison.

### Independent variables

SRH was studied based on the theoretical model previously proposed by the authors [14], considering adolescents living in an urban environment. Based on this model, the health perception of adolescents seemed to be a multidimensional indicator, defined by interactions of personal, behavioral and socio-environmental factors.

Therefore, five domains were investigated: socio-demographic, social support, lifestyle, psychological and physical health, described below.

#### 1. Socio-demographic characteristics

The following characteristics were evaluated: gender, age (11- to 13-year-olds and 14- to 17-year-olds), and monthly family income (obtained from the adult questionnaire and categorized into groups of less than five and five or more times the Brazilian minimum wage).

#### 2. Social support from family and school

Social support from family consisted of the following variables: family structure (nuclear, blended, or single-parent), the frequency of arguments in the family (none, few, or many), frequency of meals with parents (less than once a week or twice or more times a week), frequency of conversations with parents (occasionally or frequently, rarely or never), family member interest in the school life of the adolescent (no one, parent[s], or other family member), and relationship with parents (bad or good). The last variable was categorized as either bad (i.e., scores ranging from 0 to 4) or good (i.e., 5 or 6) using the following items: “My parents are always there for me when I need them”, “They make me feel loved and cared for”, “I can talk to them about any problems I might have”, “We have a lot of arguments”, “They give me the attention that I need”, and “They make me feel bad about myself”.

Regarding social support from school, the following variables were examined: satisfaction with school life (likes or does not like school), school type (public or private), and has a positive relationship with peers (considers them nice and helpful).

#### 3. Lifestyle

This domain [21] included questions about fruit consumption (at least once five days or more per week or less than five days per week), breakfast frequency (every day or never/rarely/sometimes), time spent playing video games or on the computer (less than one hour/day, two hours/day; or three hours/day or more), and physical activity over the last seven days (active: 300 minutes or more; or inactive/insufficiently active: up to 299 minutes).

Physical activity was assessed using the module based on the instrument of the Birth Cohort Study in Pelotas 1993 Brazil [21]. The physical activity module consisted of the total physical activity performed in the last seven days, combining the time and frequency of the following activities: commuting to school on foot or by bicycle, physical education classes at school, and other extracurricular physical activities [21,23]. Physical inactivity was defined as less than three hundred minutes per week of physical activity in commuting or play as currently recommended for adolescents [24].

#### 4. Psychological health

This domain was evaluated using two visual scales: life satisfaction and psychological well-being. The “Satisfaction with Life Scale” [22,25,26] uses an ascending scale from 1 to 10 on the day of interview, where the lowest value represents low life satisfaction and the highest value represents high life satisfaction. Subsequently, these responses were categorized as either positive (6 to 10) or negative (1 to 5) levels. The “Faces Scale” was used for psychological well-being [25]. This schematic instrument is composed of seven faces that refer to the prevailing mood over the two weeks prior to the interview. Psychological well-being answers were categorized as very high (face 1), high (face 2), or moderate to low (faces 3 to 7) based on a previous study [27].

#### 5. Anthropometry

Anthropometry was evaluated using body mass index (BMI), which was calculated and classified as percentiles by age group according to the World Health Organization (2007) [28]. According to this classification, a BMI below the 3^rd^ percentile was considered low; between the 3^rd^ and 85^th^ percentiles was considered adequate or normal; between the 85^th^ and 97^th^ percentiles was considered overweight; and above the 97^th^ percentile was considered obese. Age (in months) was used as a reference (years * 12 + 6 months).

### Data analysis

The adolescents were initially compared with regard to the two categories of the response variable and the explanatory variables using Pearson’s chi-square test.

Subsequently, multivariate logistic models stratified by age group were fit to the data. Explanatory variables associated with a p-value less than 0.20 in the univariate analysis were included in the multivariate analysis. Model goodness-of-fit was evaluated using the Hosmer-Lemeshow test[29]. Whenever possible, the strata homogeneity test was performed to assess the interaction between the variables [30].

To comply with the complex sample design, the weight effects were incorporated into all of the analyses using the SVY syntax within STATA 12.0. A significance threshold of 5% was used for the analyses.

### Ethical statement

The Research Ethics Committee of the Federal University of Minas Gerais approved this project (opinion no. ETIC 253/06 –extension 01/08). Adolescents participated voluntarily, and all information was considered confidential. Two informed consent forms were used, which were signed by the parents or guardians and the adolescent.

## Results

The final sample included 1,035 adolescents due to missing information. The power of the sample was calculated with a significance level of 5% and a sample error of 5% to estimate the prevalence of AAS among adolescents. The samples showed power levels of 96.0% and 95.4% for the 11- to 13-year-olds and 14- to 17-year-olds, respectively.

Of the 1,035 adolescents examined, 58.2% were 14 to 17 years old, and 52.7% were male. The overall prevalence of poor SRH was 11.2% (95% CIs = 8.7–13.6). SRH did not significantly differ by gender (p = 0.28) or income (p = 0.12). However worse rates were observed as age increased, and the greatest increase (approximately 10 percentage points) occurred among girls (from 6.9% among 11- to 13-year-olds to 16.9% among 14- to 17-year-olds). Conversely, rates increased less among boys (from 8.3% among 11- to 13-year-olds to 11% among 14- to 17-year-olds). These rates were not significantly associated with family income, even when the data were stratified by age and gender (data not shown).

Tables 1 and 2 show the results of the bivariate analyses for each age stratum. Among 11- to 13-year-olds, infrequent conversations with parents, not liking school, attendance in public school, and having low psychological well-being were associated with poor SRH. Among 14- to 17-year-olds, living in blended families, having arguments in the family, having poor peer relationships (i.e., does not consider friends as nice and helpful), consuming fruits fewer than five times/week, being physically inactive, having low psychological well-being and negative levels of life satisfaction, and being underweight or overweight (as measured by BMI) were associated with poor SRH. All comparisons were made with regard to their reference groups.

**Table 1:** Prevalence rates and confidence intervals for poor self-rated health among adolescents by socio-demographics, social support, and age group; Belo Horizonte, 2008–2009.

		11 to 13 years			14 to 17 years		
	Characteristics	(n = 435)			(n = 600)		
		%	CI 95%	P-value^#^	%	CI 95%	P-value^#^
**SOCIO-DEMOGRAPHICS**							
**Gender**	Male	8.30	2.81–13.80	0.67	10.89	7.15–14.62	0.09
	Female	6.91	3.01–10.81		16.95	10.81–23.09	
**Monthly family income**	< 5 MW	8.16	4.11–12.21	0.47	15.57	11.11–20.02	0.09
	≥ 5 MW	5.39	0.00–10.93		9.39	4.70–14.07	
**SOCIAL SUPPORT**							
**Family structure**							
	Nuclear	7.76	3.22–12.30	0.92	11.37	7.42–15.32	0.01
	Blended	5.83	0.00–16.98		27.81	14.44–41.18	
	Single-parent	8.19	2.20–14.19		13.40	7.01–19.79	
**Arguments in the family**	No arguments	2.94	0.00–6.71	0.09	6.89	2.80–10.98	< 0.0001
	Few arguments	10.12	4.72–15.52		11.52	7.09–15.95	
	Many arguments	13.70	0.00–28.06		38.83	25.82–51.86	
**Meals with parents**	≥ 2 times/week	7.48	3.52–11.44	0.78	12.57	8.91–16.24	0.24
	< 1 times/week	8.63	1.60–15.65		17.78	9.16–24.41	
**Conversations with parents**	Occasionally or frequently	5.64	2.39–8.90	0.02	12.93	9.41–16.45	0.31
	Rarely or never	17.37	4.99–29.75		18.20	7.69–28.71	
**Interest in the school life of the adolescent**	Father, mother, or both	7.52	3.61–11.42	0.19	12.24	8.35–16.13	0.46
	Another family member	5.60	0.00–11.79		13.99	4.11–23.89	
	No one shows interest	18.89	1.29–36.49		19.13	8.37–29.88	
**Relationship with parents**	Good	6.68	3.17–10.20	0.08	12.24	8.81–15.67	0.01
	Bad	18.11	10.03–35.21		29.81	13.80–45.82	
**Behind in school**	No	6.65	3.97–10.09	0.43	11.30	7.22–15.37	0.15
	Yes	10.35	8.17–19.89		16.41	10.52–22.31	
**Likes school**	Yes	6.50	3.01–9.99	0.004	12.51	8.81–16.22	0.46
	No	28.17	6.01–50.34		16.08	6.86–25.29	
**Type of school**	Private	1.01	0.00–3.01	0.01	16.22	7.85–24.60	0.41
	Public	8.66	4.60–12.74		12.49	8.63–16.35	
**Considers friends “nice and helpful”**	Yes	7.37	3.77–10.97	0.56	12.69	9.15–16.24	0.04
	No	11.70	0.00–28.54		27.28	10.42–44.14	
**Feels unsafe walking in the neighborhood**	No	10.12	4.69–15.54	0.10	11.49	7.37–15.61	0.13
	Yes	4.97	1.36–8.59		16.32	11.26–21.38	

MW—minimum wage.

^#^P-value obtained through Pearson's chi-square test.

CI—confidence interval.

**Table 2:** Prevalence rates and confidence intervals for poor self-rated health among adolescents by lifestyle, psychological health, anthropometry, and age group; Belo Horizonte, 2008–2009.

		11 to 13 years			14 to 17 years		
	Characteristics	(n = 435)			(n = 600)		
		%	CI 95%	P-value^#^	%	CI 95%	P-value^#^
**LIFESTYLE**							
Consumes fruit	≥ 5 times/week	4.55	0.12–8,98	0.17	7.19	3.72–10.67	0.002
	< 5 times/week	9.54	4.76–14.32		16.81	12.24–21.38	
Breakfast	Every day	8.32	3.42–13.21	0.49	12.90	8,68–17.13	0.53
	Sometimes, rarely, or never	6.27	2.75–9.79		15.01	9.81–20.20	
Physical activity	Active	28.23	5.92–50.54	0.07	21.27	12.55–29.98	0.06
	Insufficiently active	8.41	2.23–14.58		13.26	8.94–17.57	
	Inactive	6.73	1.93–11.52		9.24	3.48–14.99	
Watches television	≤ 1 hour/day or not at all	9.56	1.37–17.74	0.81	9.53	4.94–14.11	0.20
	2 hours/day	6.97	0.00–14.22		12.65	4.82–20.48	
	≥ 3 hours/day	7.09	3.05–11.13		16.30	11.15–21.46	
Plays video games or uses the computer	≤ 1 hour/day or not at all	7.02	2.69–11.34	0.90	16.34	2.70–11.34	0.10
	2 hours/day	8.78	0.00–17.98		6.10	0.00–17.98	
	≥ 3 hours/day	8.55	1.61–15.49		12.80	1.61–15.50	
**PSYCHOLOGICAL HEALTH**							
Psychological well-being	Very high	3.83	0.08–7.59	0.04	6.47	1.93–11.01	0.0009
	High	5.69	0.00–12.34		10.42	5.41–15.43	
	Low	14.70	6.76–22.64		20.95	14.72–27.19	
Life satisfaction	Positive	7.42	3.53–11.31	0.90	11.14	7.74–14.53	0.0001
	Negative	6.95	0.89–13.01		29.70	18.86–40.55	
**ANTHROPOMETRY**							
Body mass index	Normal weight	5.88	1.73–10.02	0.27	10.42	7.19–13.64	0.004
	Underweight	11.74	0.00–29.76		34.33	10.15–58.51	
	Overweight	12.45	4,65–20.24		21.10	10.80–31.40	

^#^P-value obtained through Pearson's chi-square test.

CI—confidence interval.

Table 3 shows the results of the multivariate model. Only infrequent conversation with parents (odds ratio [OR] = 3.4; 95% confidence interval [CI] = 1.1–11.2) was associated with poor SRH among 11- to 13-year-olds. Among 14- to 17-year-olds, the following characteristics remained significantly associated with poor SRH after fitting the final model: blended family structure (OR = 2.82; 95% CI = 1.23–6.51); many arguments in the family (OR = 8.21; 95% CI = 3.27–20.58), low fruit consumption (OR = 2.44; 95% CI = 1.25–4.75), underweight status (OR = 6.74; 95% CI = 1.79–25.45), overweight status (OR = 2.68; 95% CI = 1.26–5.83), and life dissatisfaction (OR = 2.85; 95% CI = 1.39–5.83). Although gender was not significantly associated with poor SRH for either age strata, it was maintained in both final models as a control variable.

**Table 3:** Multivariate logistic regression for poor self-rated health among adolescents; Belo Horizonte, 2008–2009.

		Age group	
Characteristics		11 to 13 years	14 to 17 years
		OR (95% CI)	OR (95% CI)
Gender	Male	1.0	1.0
	Female	1.05 (0.38–2.92)	0.60 (0.31–1.17)
**SOCIAL SUPPORT**			
Family structure	Nuclear	-	1.0
	Blended		**2.82 (1.23–6.51)**
	Single-parent		1.34 (0.63–2.88)
Arguments in the family	No arguments	-	1.0
	Few arguments		1.94 (0.83–4.50)
	Many arguments		**8.21 (3.27–20.58)**
Conversations with parents	Occasionally or frequently	1.0	-
	Rarely or never	**3.49 (1.15–10.68)**	
**LIFESTYLE**			
Consumes fruit	≥ 5 times/week	-	1.0
	< 5 times/week		**2.44 (1.25–4.75)**
**PSYCHOLOGICAL HEALTH**			
Life satisfaction	Positive	-	1.0
	Negative		**2.85 (1.39–5.83)**
**ANTHROPOMETRY**			
BMI	Normal weight	-	1.0
	Underweight		**6.74 (1.79–25.45)**
	Overweight		**2.68 (1.26–5.73)**
	**P-value** *	0.6248	0.4672

The variables included in the model were those whose p-values were less than 0.20 in the bivariate analysis (see Tables 1 and 2).

OR—odds ratio.

CI—confidence interval.

*Hosmer-Lemeshow test (goodness-of-fit of the model).

## Discussion

We aimed to determine the prevalence of poor SRH within two age groups of adolescence using a household survey. We found that 11% of adolescents perceived their health as poor, and this perception deteriorated with age among males and females. However, this decrease in SRH was more pronounced (and significant) among girls. Family environments characterized by weak social ties (i.e., lack of warmth or fragile relationships) were the only quality associated with negative health assessments among younger adolescents. However, among older adolescents, domains related to lifestyle and psychological and physical health were associated with poor SRH.

A comparison of our results with previous studies conducted among Brazilian adolescents shows that the current prevalence of poor SRH was lower than the 15.8% observed among 2,859 students aged between 14 and 19 years in Paraíba [15] and the 14.4% identified among 5,028 students aged between 15 and 19 years in Santa Catarina [9]. Furthermore, a study conducted across 43 countries in Europe and North America revealed that 11%, 15%, and 18% of 11-year-olds, 13-year-olds, and 15-year-olds, respectively, had poor SRH [31].

The between-study differences in the prevalence of poor SRH might be due to the absence of an international standard for response options, the way in which the SRH measure is categorized, the location where the adolescent completes the questionnaire (i.e., home or school environments), the way in which the questionnaire is completed (i.e., self-completed or via face-to-face interview), the source of information (i.e., randomly selected individuals or third parties), differences in the placement of the question within the survey, aspects of study design and model fit [32].

Nonetheless, the dissimilarities could also be related due to the age groups studied, as confirmed in the literature; namely, the prevalence of poor SRH increases with age. The proportion of 11- to 13-year-old males with poor health perceptions (8.3%) was greater than that of their female counterparts (6.9%); among 14- to 17-year-olds, the proportion of males (10.9%) with poor SRH was lower than that among females (16.9%). These findings may have support by what has been observed in studies of adults, where the prevalence of poor SRH is significantly greater among females [3,33] than males. Adolescent girls may become increasingly preoccupied with their health as they age, with similar health perception patterns as adult women, perhaps beginning at 14 to 17 years old.

Regarding the determinants of poor SRH, importantly, family characteristics were associated with adolescents’ self-perceptions of health and well-being regardless of age. This finding suggests that family support and relationships are essential throughout adolescence. According to Vilhjalmsson, parental social support is more important for SRH than other types of support because of the various effects that parents exert on the health-related behaviors of their adolescent offspring [34].

Among 11- to 13-year-olds, SRH was only associated with family support, whereas SRH was associated with broader constructs involving social support, lifestyle, psychological health, and anthropometry among older adolescents. This finding suggests that, with maturation (as defined by age in the present study), adolescents are no longer shaped exclusively by parental relationships; rather, more complex issues such as lifestyle and life satisfaction gain importance. The increase in problems that young people face and the changes in the way that they perceive themselves and their health statuses might enlighten this finding [35]. Family support also determined the negative health perceptions of 14- to 17-year-olds. However, other family environment constructs, such as family structure and frequency of arguments, were associated with SRH among this age group. Fourteen- to 17-year-olds living in blended families were approximately three times more likely to have poor SRH than those living in nuclear families. These findings match those of international studies showing that adolescents who live in single-parent and blended families have poor SRH [7,36,37]. However, some authors have argued that this effect is not direct and is most likely mediated by the quality of the family’s interactions and their financial situation [36]. According to Heard, the influence of family structure on adolescents might be explained by the parent-adolescent relationship because this bond is weakened in single-parent families. Hence, family cohesion might be lower in blended families [36].

Report of arguments was another factor strongly associated with low SRH, and this situation may cause family conflicts. Although the CIs were large because of small numbers, older adolescents who report that many arguments occur in their domestic environment were 8.2 times more likely to perceive their health as worse than those who did not report arguments in the family. According to Herrenkohl et al. (2009), family conflict can lead to depressive symptoms and stressful events in adulthood; moreover, this situation tends to increase with age among 14- to 18-year-olds [38].

With regard to the lifestyle domain, 14- to 17-year-olds who reported consuming fruit less than five times a week were 2.4 times more likely to display poor SRH than those who consumed more fruit per week. This finding suggests the importance of healthy eating habits for the health perceptions of this age group. We did not find previous studies that evaluated this factor related to SRH among adolescents, which reinforces the need for broader studies that evaluate the health perception of adolescents.

Psychological health was only associated with SRH among 14- to 17-year-olds: those who had less life satisfaction had poor SRH. The importance of psychological well-being with regard to the SRH of adolescents has been observed by studies that have addressed subjective health assessment using single-item measures [6,35,37].

With regard to BMI, both 14- to 17-year-olds who were underweight and those who were overweight had low SRH. Some studies of adolescents have reported an association between excess weight and poor SRH [8,15,39]. The study conducted with 14- to 19-year-old students from João Pessoa showed that overweight adolescents were three times more likely to perceive their health in a negative way than those who were not overweight [15]. According to these authors, older adolescents perceive the negative implications to their health caused by their overweight or obese statuses. The media emphasizes the importance of body weight, and adolescents adopt socially determined “standards” that reinforce the adverse effects to their health caused by excess weight that might decrease SRH among adolescents [15].

In summary, underweight and overweight as measured by BMI, low fruit consumption, and life dissatisfaction significantly predicted low SRH among 14- to 17-year-olds. All of these findings may suggest that concerns that are usually perceived as belonging to the adult world [40] may also affect older adolescents.

Certain limitations of the present study must be considered, including its cross-sectional nature, which does not allow us to establish causality. Regarding the use of SRH, as a single-item measure, it is based on its recognition as a valid indicator, a strong predictor of mortality and its high correlation with objective health measures. However, some authors have questioned the use of this indicator for international comparisons and among subpopulations, arguing that different understandings of health are influenced by cultural and social factors, and the results are not always consistent with objective health indicators [40,41].

In addition, researching SRH poses difficulties because of the need for extensive information that enables researchers to control for potential confounders [2,42]. Although the present study included information regarding different aspects of adolescent health and its determinants, it did not investigate reported morbidity and only objectively measured participants’ weights and heights. It is also worth noting that variables were not included evaluating the use/frequency of health services and type health services, as these variables have not been investigated among adolescents in the “Health in Beagá Study”. Thus, a more in-depth analysis regarding the physical health dimensions of SRH was limited. The location where adolescents complete the questionnaire might influence their responses, thereby leading to the omission of certain behaviors at risk for social judgment and criticism or the exaggeration of certain answers to please the interviewer (i.e., the “social desirability bias”) [43]. The self-administered questionnaire was designed to minimize these possibilities while trying to ensure the privacy of the adolescents in their home environments. We also sought to facilitate the administration of the instrument and to minimize interviewer interference during data collection. However, adolescents might have difficulties interpreting and understanding the questions, thereby leading to some inadequate questionnaire completion.

The data from the present study referred to two out of the nine sanitary districts of the city; thus, the data may not represent the entire population of Belo Horizonte. A previous study [14] showed that some of the data collected in the “Health in Beagá Study” are similar to those collected by the National Adolescent School-based Health Survey (Pesquisa Nacional de Saúde do Escolar—PeNSE) [44] conducted among students from Belo Horizonte during the same year by the Brazilian Institute of Geography and Statistics (Instituto Brasileiro de Geografia e Estatística; IBGE), which suggests that these data have external validity.

We chose the method of dichotomizing a five-question SRH questionnaire because it is often collapsed into a dichotomous variable of good versus less than good health when it is used as a dependent variable. The collapsing of categories of a categorical variable has been discussed in the statistical literature [45]. It is recognized that dichotomization, while valid, involves loss of information and may lead to a reduction in efficiency in the statistical analysis under consideration. Manor et al. (2000) sought to establish whether results for the dichotomous outcome differ from those obtained with alternative approaches based on SRH as an ordered categorical variable [45]. They found only small differences in power and efficiency, and the results obtained using the logistic regression approach were similar to those obtained using the other methods. Thus, given the simplicity and wide use of logistic regression, particularly in epidemiology, these authors supported this practice within the context of a large sample size. In our study, only 1.4% of adolescents rated their health as poor or very poor, reinforcing the need for collapsing the data into a dichotomous categorization.

The association between certain variables (e.g., many arguments in the family and underweight status) and poor SRH exhibited ORs with large CIs due to the small sample sizes of these categories. Therefore, these results should be interpreted with caution.

Importantly, several strategies were used to avoid bias during data collection, such as previous and exhaustive tests of the instruments, the adoption of standardized procedures and equipment, the extensive training of field personnel, and various attempts to raise community awareness to encourage study participation. Thus, we sought the quality of information and the internal validity of the study.

Our findings may contribute to studies involving adolescents either in the public health field or for pediatric clinical practice. Reinforced by the literature, the present study demonstrates the merits of using a single-item measurement to assess adolescent health. Epidemiological studies providing information on the SRH of adolescents and its possible determinants at a population level are relatively scarce in the literature [2,3,4,5,9]. The age differences observed herein with regard to health perception suggest the need to develop differentiated interventions based on participant age to promote the health and well-being of youths. In addition, the results recommend that the balance between programs that aim to improve the physical and psychosocial health of adolescents must be considered, as does the need to ensure that policies are supported by action plans based on a detailed knowledge of the maturation processes of adolescents [31].

Public policies aimed at this age group can be planned and developed to act on the predictors of SRH. Examples of these policies have been health promotion programs that encourage physical activity, maintaining body weight, healthy eating and to encourage family life and life with their peers through public spaces and leisure areas that ensure healthy, pleasurable social environments.

As an example of these programs, we can cite the HIPPY (Home Instruction Program for Preschool Youngsters) and the Brighter Futures programs, both developed in England. HIPPY, which was not English originally, advocates the encouragement of positive interactions between parents and children, and researchers involved in the project believe that the bond formed between parents and children in childhood reverberate in adolescence and adulthood [46]. Brighter Futures acts with children and young people and encompasses aspects beyond literacy, including actions that improve the physical health of the child and their behavior, emotional health, social development and development of job skills [47].

In Brazil, we can mention the School Health Program (Programa Saúde na Escola—PSE), an intersectoral policy of Health and Education that aims to promote health and to contribute to the creation of conditions for the integral formation of students. The PSE works with actions that involve assessment of nutritional status; promoting adequate and healthy food; promotion of corporal practices, physical activity and leisure; environmental health; oral health; mental health; and eye health [48].

In summary, the current research revealed that adolescents’ SRH might be influenced by issues that extend beyond physical health. Furthermore, family social support strongly predicts SRH throughout adolescence, indicating the importance of the family during this developmental stage. Parental support might improve the SRH of youths [32], and the family retains its significance because it continues to play a central role throughout the development of its adolescent members, with individual functions at each step [49].

For these reasons, it becomes imperative to invest in orientation programs that provide parents with tools to more adequately manage their adolescent children. In addition, an understanding of the single-item SRH measure that is often used in surveys is essential for its use in epidemiological studies and serves as a basis for public policies aimed at adolescent well-being.
